# Personalizing fall fear prevention in knee osteoarthritis: an interpretable prediction framework via IGKSO synchronous optimization and SHAP-based risk stratification

**DOI:** 10.3389/fpubh.2026.1749921

**Published:** 2026-02-26

**Authors:** Min Yin, Wenjing Fang, Yuanna Cheng, Yanru Feng

**Affiliations:** 1 Orthopedics Department, The 945th Hospital of the Joint Logistics Support Force of the Chinese People's Liberation Army, Yaan, Sichuan, China; 2 Outpatient Department, General Hospital of Western Theater Command, Chinese People's Liberation Army, Chengdu, Sichuan, China

**Keywords:** artificial intelligence, concern about falling, forecasting, knee osteoarthritis, machine learning

## Abstract

**Objective:**

To construct a concern about falling (CAF) prediction model for patients with knee osteoarthritis (KOA) based on synchronous optimization.

**Methods:**

A total of 541 patients with KOA admitted to two hospital from September 2021 to September 2023 were selected. CAF was evaluated using the Falls Efficacy Scale-International (FES-I). Patients were divided into a CAF group (*n* = 360, FES-I ≥ 28 points) and a no CAF group (*n* = 181, FES-I < 28 points). 80% of the data (433 cases) were used as the training set, and the remaining 20% (108 cases) were used as the test set. An improved swarm intelligence algorithm was used for feature selection and hyperparameter optimization. The selected variables were further analyzed by Shapley Additive exPlanation (SHAP) interpretable method.

**Results:**

In the training set, the maximum F1 score of the improved synchronous optimization machine learning model was 0.8842, and the area under the curve reached 0.9451. In the test set, the maximum F1 score of the improved synchronous optimization machine learning model was 0.8589, and the area under the curve reached 0.9315. Eight variables were selected based on the improved synchronous optimization machine learning model, including Timed Up-and-Go (TUG) time, Western Ontario and McMaster Universities Osteoarthritis (WOMAC) pain score, Hospital Anxiety and Depression Scale (HADS) anxiety score, knee extensor moment, age, sex, Kellgren-Lawrence (KL) grade, and Body mass index (BMI). Critically, SHAP analysis demonstrated triadic interactive effects among key risk indicators, revealing that older adult female patients with concurrent TUG time >14 s, HADS-anxiety scores >10, and high WOMAC pain scores constituted the peak-risk cohort amplified through bio-psycho-social interactions.

**Conclusion:**

This study validated a multimodal predictor model for CAF in knee osteoarthritis (KOA) patients through a machine learning framework, revealing synergistic mechanisms among biomechanical, psychological, and social dynamics, with specific risk stratification for high-risk populations such as older adult females to guide clinical practice.

## 1 Introduction

Knee osteoarthritis (KOA), a chronic degenerative joint disease characterized by pain, stiffness, and functional impairment, imposes significant clinical and economic burdens globally, with prevalence exceeding 50% in adults ≥60 years (1, 2). The knee's role as the primary weight-bearing joint makes it uniquely vulnerable to mechanical stressors, leading to pathological changes like cartilage degeneration and impaired neuromuscular control (3, 4). This often triggers a debilitating cycle of reduced mobility, muscle deconditioning, and heightened fall risk (5, 6). Crucially, concern about falling (CAF) – distinct from actual falls – is increasingly recognized as a prevalent and independent predictor of functional decline in KOA, affecting approximately 40% of patients and substantially diminishing quality of life and healthcare resource utilization (7).

Current strategies for identifying high CAF risk face significant limitations. While instruments like the Falls Efficacy Scale-International (FES-I) provide definitive assessment and serve as a clinical gold standard (8, 9), their administration requires dedicated resources, hindering routine screening. Machine learning (ML) models show promise in handling complex clinical datasets – incorporating variables like Western Ontario and McMaster Universities Osteoarthritis (WOMAC) stiffness scores [joint limitation severity (10)] and Hospital Anxiety and Depression Scale (HADS) anxiety metrics [psychological distress (11)] – yet frequently lack interpretability, functioning as inaccessible “black-boxes”(12). Although hybrid approaches using wearable sensors improve biomechanical assessment accuracy, their clinical application is limited by unresolved algorithmic bias in translating sensor data to psychological states of concern and by inadequate integration of dynamic risk factors like illness duration or functional status which modulate CAF (13–15).

To address these challenges, our study pioneers an integrated computational framework that synergizes clinical workflow needs with optimized algorithm design. We developed an enhanced swarm intelligence optimization method enabling synchronous feature selection and hyperparameter tuning to improve model robustness and interpretability. This was embedded within an XGBoost ensemble algorithm. Most importantly, we created an interactive visualization interface to transform algorithmic outputs into actionable clinical insights. This approach shifts focus from retrospective documentation to prospective prevention, bridging the implementation gap for computational tools in KOA management by providing a practical pre-screening solution based on routinely available clinical data.

## 2 Data and methods

### 2.1 General information

**Figure 1:**
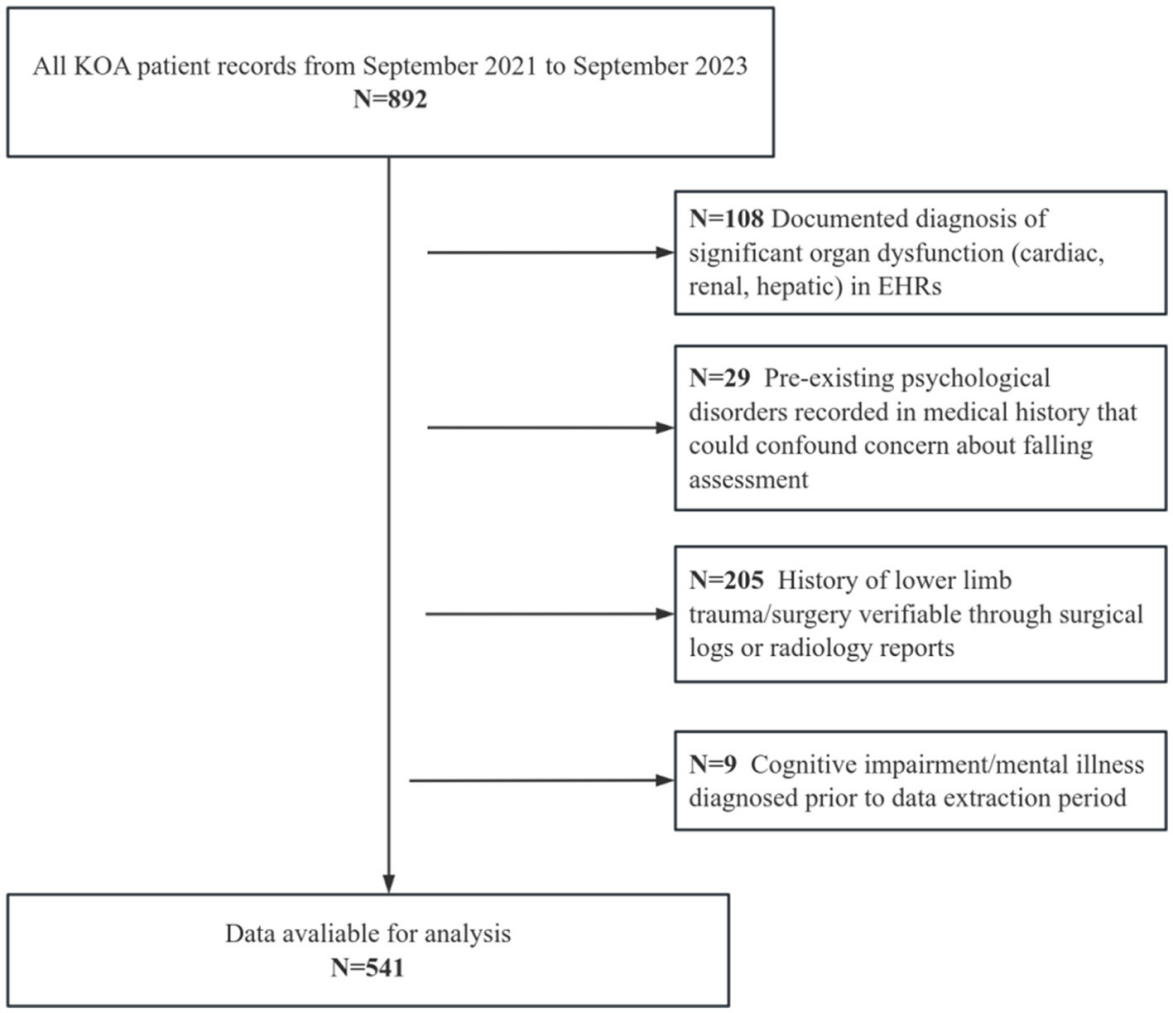
Participant flowchart.

Our study adopted a retrospective design, and analyzed pre-existing clinical data from deidentified electronic health records at the 945th Hospital of the Joint Logistics Support Force of the Chinese People's Liberation Army and General Hospital of Western Theater Command, Chinese People's Liberation Army. No new recruitment occurred. The data for these patients were accessed for research purposes from September 2021 to September 2023, serving as the study population. The inclusion and exclusion criteria are as follows: (1) Inclusion criteria: ① Conforming to the guidelines and standards published by the International Society of Arthritis and Rheumatism (16). ② Audible joint sounds during movement, bone tenderness, knee joint examination indicating osteophyte formation, absence of significant synovial warmth, and radiographic severity was staged via Kellgren-Lawrence (KL) classification, with inclusion limited to patients with KL grade 2 (definite osteophytes, possible joint space narrowing) or grade 3 (multiple osteophytes, definite joint space narrowing). ③ Possessed complete electronic health records (EHRs) including baseline WOMAC scores. (2) Exclusion criteria ① Documented diagnosis of significant organ dysfunction (cardiac, renal, hepatic) in EHRs. ② Pre-existing psychological disorders recorded in medical history that could confound CAF assessment. ③ History of lower limb trauma/surgery verifiable through surgical logs or radiology reports. ④ Cognitive impairment/mental illness diagnosed prior to data extraction period. The details of the participants are shown in Figure 1.

CAF among patients was assessed using the FES-I (17), Patients were stratified using the FES-I cut-off of 28, validating clinically significant CAF (moderate-to-high concern) vs. low concern, consistent with standardized classifications, with patients categorized into a CAF group (*n* = 360, FES-I ≥ 28) and a no CAF group (*n* = 181, FES-I < 28) (18). This study obtained approval from the Ethics Committee of the 945th Hospital of the Joint Logistics Support Force of the Chinese People's Liberation. Due to the retrospective nature of this study, the Ethics Committee exempted the requirement for informed consent from the patients (Ethical Application Ref: 202310039). Our study was conducted in accordance with relevant guidelines/regulations and in accordance with the provisions of the Declaration of Helsinki.

### 2.2 Methods

#### 2.2.1 Data collection

We extracted the following information from electronic health records: (1) Demographic and basic characteristics: age, sex, body mass index (BMI), disease duration, and employment status (employed, unemployed, retired); (2) Objective functional assessment metrics: To enhance model objectivity, we included key motor function indicators: concentric peak moment of knee extensors measured by a hand-held dynamometer (unit: Nm/kg, reflecting muscle strength), Timed Up-and-Go (TUG) test time (unit: seconds, assessing dynamic balance and mobility), and gait speed during the 10-meter walk test (unit: m/s, evaluating walking function); (3) Clinical and imaging characteristics: Radiographic severity was staged using the KL classification, while information on bone marrow edema and joint effusion was extracted from available knee MRI reports and recorded dichotomously; (4) Symptom assessment: The WOMAC (19) was used to evaluate knee osteoarthritis symptoms, collecting its three subscale scores for pain, stiffness, and physical functional limitations; (5) Psychological assessment: Anxiety status was assessed using the HADS (20). All collected variables were used for subsequent feature selection and model construction. Data were extracted from complete records; no missing values existed in the final analysis dataset, thus requiring no imputation.

#### 2.2.2 Improved swarm intelligence algorithm

**Figure 2:**
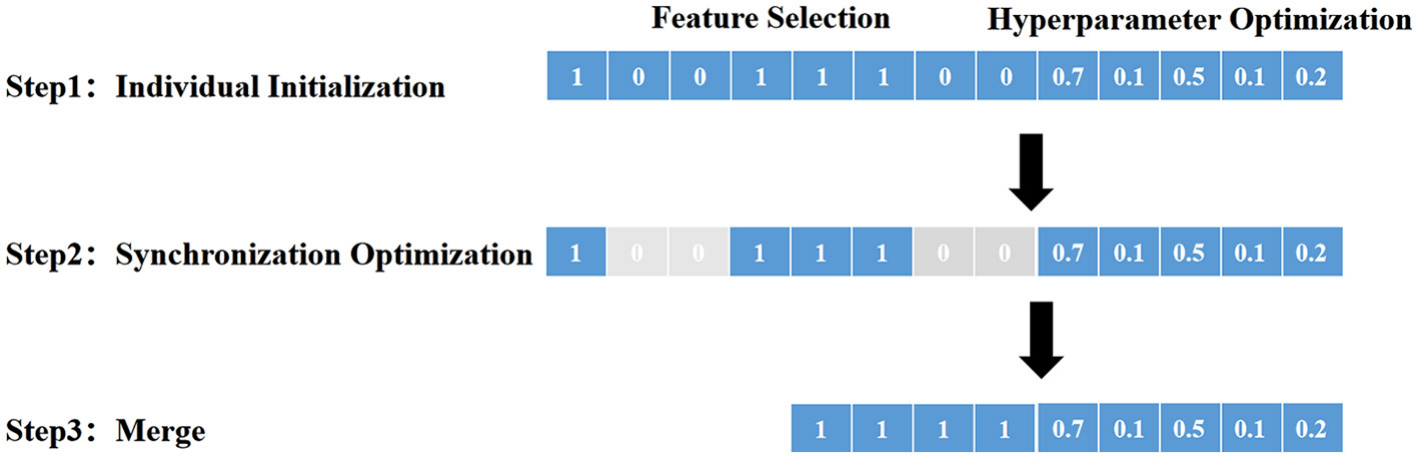
Synchronous optimization of machine learning principle diagram. (1) Individual Initialization: In this step, the initial population is created and set as the starting point of the swarm intelligence algorithm, where each individual represents a set of feature combinations and hyperparameter configurations. (2) Synchronization Optimization: The swarm intelligence algorithm simultaneously conducts feature selection and hyperparameter optimization. Through collaboration and competition, individuals exchange information and gradually optimize features and parameters to enhance model performance. (3) Merge: Finally, after multiple rounds of iterative optimization, the results from individuals are merged to form the optimal feature combinations and parameter configurations, used for training the machine learning model, thus achieving more accurate and efficient prediction and decision-making processes.

To address the intricate optimization requirements of simultaneous feature selection and hyperparameter tuning in our study, an enhanced swarm intelligence algorithm—the Improved Genghis Khan Shark Optimizer (IGKSO)—was developed, building upon the foundational Genghis Khan Shark Optimizer (GKSO) which mimics the predatory behavior of Genghis Khan sharks for numerical optimization (21, 22). Critical algorithmic refinements were implemented to bolster its global optimization capabilities: Bernoulli chaotic mapping was incorporated during population initialization to amplify randomness, while spiral flight mutation was introduced to strengthen global search dynamics. The optimization efficacy of IGKSO was rigorously validated through simulations on 23 standardized benchmark test functions, encompassing both unimodal (U) and multimodal (M) categories with varying dimensions, complexities, and search space boundaries. Unimodal functions evaluated local exploitation capabilities through single-optima landscapes, whereas multimodal functions assessed global exploration capacity across multiple optima. Under consistent experimental conditions (population size = 30, iterations = 200, 30 independent repetitions), average convergence curves quantitatively demonstrated performance improvements post-enhancement. Leveraging these verified capabilities, IGKSO was deployed for synchronous machine learning optimization—an integrated methodology that concurrently executes feature selection and hyperparameter configuration. This co-adaptive approach dynamically explores feature subspaces while tuning parameters in response to problem-specific characteristics, thereby efficiently identifying synergistic feature-parameter combinations that maximize model performance and generalization ability. The methodology (detailed in Figure 2) optimizes computational efficiency, elucidates feature-performance relationships, and delivers robust solutions for complex real-world challenges through three core phases: (1) Individual Initialization generating candidate solutions representing feature-parameter sets; (2) Synchronization Optimization where swarm collaboration/competition refines selections; (3) Merge of optimized configurations for final model training and predictive deployment.

#### 2.2.3 Machine learning model construction and explainability analysis

Firstly, during data partitioning, stratified random sampling was applied based on the outcome variable to divide the entire dataset into a training set (*n* = 433) and an independent testing set (*n* = 108) at a 4:1 ratio, ensuring proportional distribution of outcome classes in both the original cohort and subsets. We analyzed differences in features selected by the synchronous optimization model between patient groups and outputted weight rankings per feature. Four supervised machine learning algorithms were implemented as base learners: Logistic Regression (LR): Selected for its interpretability and efficient linear classification capability; Back Propagation Neural Network (BP): Incorporated for universal function approximation capacity; Support Vector Machine (SVM): Utilized for its robustness in high-dimensional spaces using kernel tricks; XGBoost: Employed as a gradient-boosted tree ensemble method optimized for computational efficiency. This selection covers distinct learning paradigms (linear models, neural networks, kernel methods, ensemble methods) to comprehensively evaluate our synchronous optimization framework while representing established approaches in clinical prediction research. The names and range standards for hyperparameter optimization during each learning phase are shown in Table 1. The feature selection process occurred through synchronous optimization using IGKSO. From an initial pool of clinically relevant variables spanning demographics, functional assessments, and psychological metrics, the algorithm identified optimal feature-hyperparameter combinations. The selected variables maximized predictive performance while minimizing overfitting. Following feature selection by the IGKSO-optimized model, univariate and multivariable logistic regression analyses were conducted for clinical validation of the algorithmically identified variables. Shapley Additive Explanations (SHAP) interpretability analysis: The deconstruction of model logic strictly followed the SHAP framework based on coalition game theory. By calculating Shapley values, we performed numerical attribution of feature contribution to individual predictions, and comprehensively applied summary plots to reveal the ranking of global feature importance. Additionally, waterfall plots, decision plot, and force plots were used to intuitively visualize the explanatory process of specific predictions.

**Table 1:** Hyperparameter optimization table for base Learners.

**Learner**	**Hyperparameter**	**Type/range**	**Description**
Logistic regression (LR)	penalty	[‘1′,'2′]	Regularization type (L1/L2 norm)
	C	[0.001, 100] (log-uniform)	Inverse regularization strength; smaller = stronger constraint
	max_iter	[50, 500] (integer)	Maximum iterations for convergence
BP neural network (BP)	hidden_layer_sizes	e.g., (50), (100), (50,50)	Architecture of hidden layers
	activation	[‘relu', ‘tanh', ‘logistic']	Activation function
	learning_rate_init	[0.0001, 0.1] (log-uniform)	Initial learning rate
	alpha	[0.0001, 0.1] (log-uniform)	L2 regularization coefficient
	batch_size	[16, 128] (integer)	Mini-batch size for gradient updates
Support vector machine (SVM)	C	[0.1, 100] (log-uniform)	Misclassification penalty coefficient
	kernel	[‘linear', ‘rbf', ‘poly']	Kernel function type
	gamma	[0.0001, 10] (log-uniform)	Kernel width for RBF/poly (impact on non-linearity)
	degree	(2, 5) (integer)	Polynomial degree (activated when kernel = ‘poly')
XGBoost	learning_rate	[0.01, 0.3]	Boosting step shrinkage (controls overfitting)
	max_depth	(3, 12) (integer)	Maximum tree depth
	min_child_weight	(1, 10) (integer)	Minimum sum of instance weight in a leaf node
	subsample	[0.5, 1.0]	Row subsampling ratio
	colsample_bytree	[0.5, 1.0]	Feature subsampling ratio per tree
	reg_alpha	[0, 1]	L1 regularization term (induces feature sparsity)
	reg_lambda	[0, 2]	L2 regularization term

### 2.3 Statistical analysis

Traditional statistical analysis was performed using IBM SPSS Statistics 25.0 (IBM Corp., Armonk, NY, USA). Categorical data were presented as n (%) and compared using the χ^2^ test. Normally distributed continuous data were expressed as mean ± standard deviation and analyzed with the t-test, with statistical significance defined as *P* < 0.05 for two-tailed tests. Machine learning workflows—including IGKSO-based feature selection and hyperparameter optimization, XGBoost modeling, and SHAP analysis—were implemented in MATLAB 2025b (The MathWorks, Natick, MA, USA) with Machine Learning Toolbox (v15.1). Model evaluation metrics included sensitivity (SEN), precision (PRE), specificity (SPE), accuracy (ACC), F1-Score (F1), receiver operating characteristic-area under the curve (ROC-AUC), and precision-recall-area under the curve (PR-AUC). All metrics range from 0 to 1, with higher values indicating superior performance. Decision curve analysis (DCA) was applied to quantify the model's clinical utility by calculating the net benefit (NB) across varied threshold probabilities. This enabled validation of the model's effective decision-assistance range through comparative analysis between NB values and conventional intervention strategy reference lines. Meanwhile, calibration curves accompanied by Brier scores (where lower values indicate better prediction accuracy) were utilized to assess probabilistic prediction precision.

## 3 Results

### 3.1 Baseline characteristics of different datasets

A total of 541 patients were included in our study. Among them, 360 patients (66.54%) experienced CAF. The clinical characteristics of patients in the training set (*n* = 433) and the testing set (*n* = 108) are compared below (Table 2). The results demonstrate no statistically significant differences (*p* > 0.05) in any of the clinical characteristics between the two datasets.

**Table 2:** Comparison of clinical characteristics between training set and testing set.

**Variables of interest**	**Training set (*n* = 433)**	**Testing set (*n* = 108)**	**Statistics value**	***P***
Age (Year), Mean ± SD	64.52 ± 13.23	65.12 ± 11.46	0.433	0.666
BMI (kg/m^2^), Mean ± SD	23.98 ± 3.15	24.12 ± 3.42	0.406	0.685
Duration of illness (Year), Mean ± SD	15.82 ± 4.62	16.11 ± 4.54	0.586	0.558
**Sex**, ***n*** **(%)**				
Male	130 (30.02)	34 (31.48)	0.087	0.768
Female	303 (69.98)	74 (68.52)		
**Employment status**, ***n*** **(%)**				
On the job	81 (18.71)	21 (19.44)	0.011	0.915
Unemployment	121 (27.94)	30 (27.78)		
Retired	231 (53.35)	57 (52.78)		
**Occupation (*****n*****)**, ***n*** **(%)**				
Farmer	227 (52.42)	57 (52.78)	0.004	0.948
White collar	184 (42.49)	45 (41.67)		
Freelance work	22 (5.09)	6 (5.55)		
**Degree of education**, ***n*** **(%)**				
Primary school and below	155 (35.80)	38 (35.19)	0.014	0.906
Middle school	234 (54.04)	59 (54.63)		
College or above	44 (10.16)	11 (10.18)		
**Mode of living**, ***n*** **(%)**				
Live alone	41 (9.47)	10 (9.26)	0.037	0.847
Living with a spouse	232 (53.58)	58 (53.70)		
Living with children	61 (14.09)	16 (14.81)		
Live with the whole family	99 (22.86)	24 (22.23)		
**KL classification**, ***n*** **(%)**				
Grade 2	266 (61.43)	63 (58.33)	0.327	0.567
Grade 3	167 (38.57)	45 (41.67)		
MRI bone marrow edema, *n* (%)	189 (43.65)	45 (41.67)	0.138	0.710
MRI joint effusion, *n* (%)	156 (36.03)	42 (38.89)	0.305	0.581
Knee extensor moment (Nm/kg), Mean ± SD	1.21 ± 0.38	1.19 ± 0.36	0.494	0.621
TUG time (seconds), Mean ± SD	12.45 ± 3.67	12.88 ± 3.45	1.102	0.271
Gait speed (m/s), Mean ± SD	0.92 ± 0.21	0.90 ± 0.22	0.877	0.381
WOMAC pain score, Mean ± SD	5.49 ± 2.24	5.53 ± 2.17	0.167	0.867
WOMAC stiffness score, Mean ± SD	2.15 ± 0.49	2.17 ± 0.43	0.388	0.698
WOMAC Functional Disability score, Mean ± SD	22.53 ± 9.25	23.18 ± 10.23	0.639	0.523
HADS anxiety score, Mean ± SD	10.21 ± 3.52	10.35 ± 3.47	0.371	0.711

Values are expressed as *n* (%) for categorical variables and mean ± standard deviation for continuous variables. BMI, Body mass index; KL, Kellgren-Lawrence grade; TUG, timed up and go; WOMAC, western ontario and Mcmaster universities osteoarthritis index; HADS, hospital anxiety and depression scale.

### 3.2 Performance testing of improved swarm intelligence algorithm

**Figure 3:**
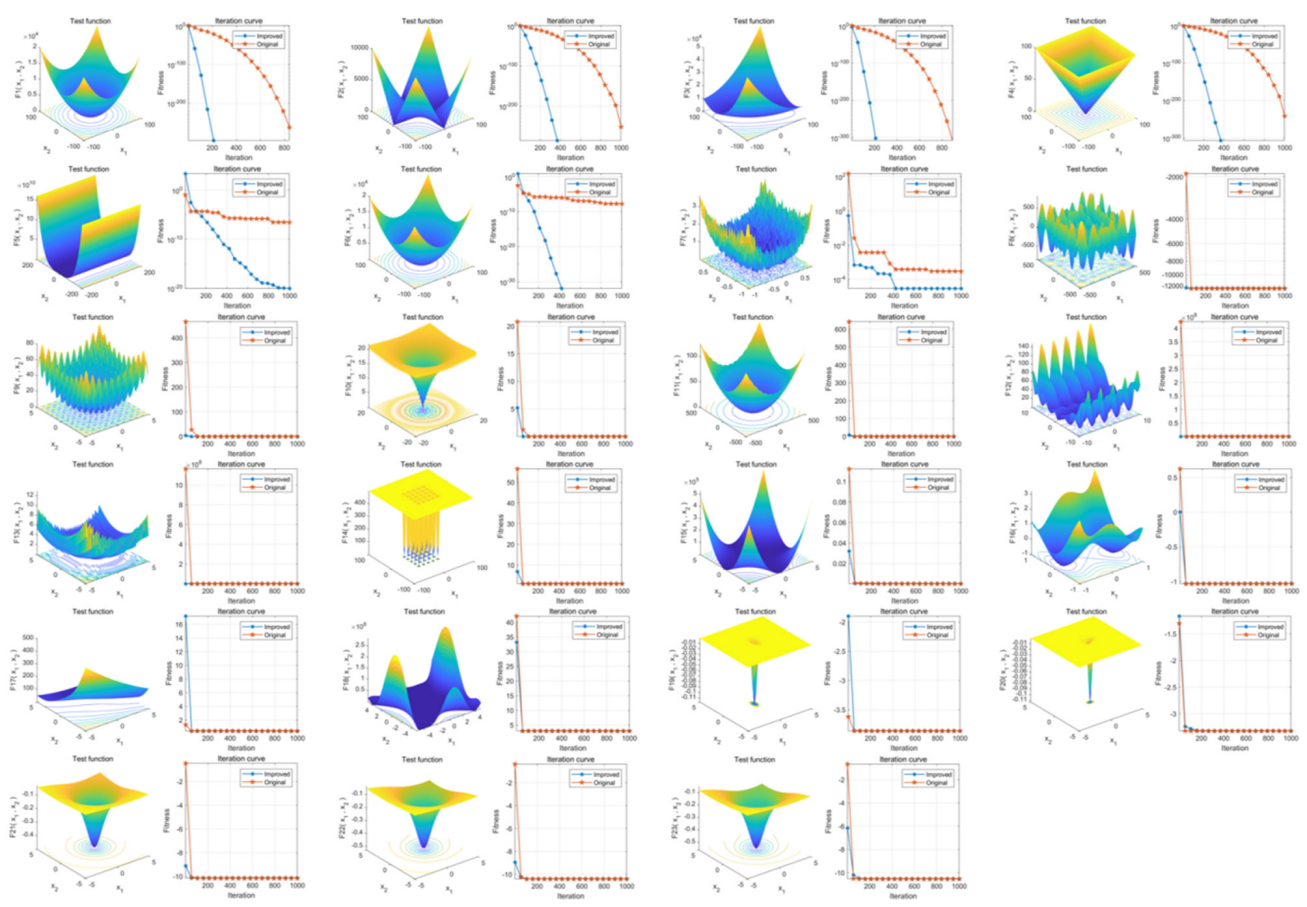
Comparison of Optimization Capability Before and After Improvement in IGKSO. The three-dimensional surface plots in the figure demonstrate the two-dimensional search space for each benchmark function. The convergence curves illustrate the convergence trends of the first-dimensional first solution for each benchmark function, and compare the trends between GKSO and IGKSO. The red convergence curve corresponds to the original GKSO algorithm, while the blue convergence curve corresponds to the improved IGKSO algorithm.

The results indicate that the overall convergence and global optimization capability of our IGKSO have significantly surpassed those of the original GKSO algorithm. This enhancement in global optimization performance is visually demonstrated in Figure 3.

### 3.3 Model training

**Figure 4:**
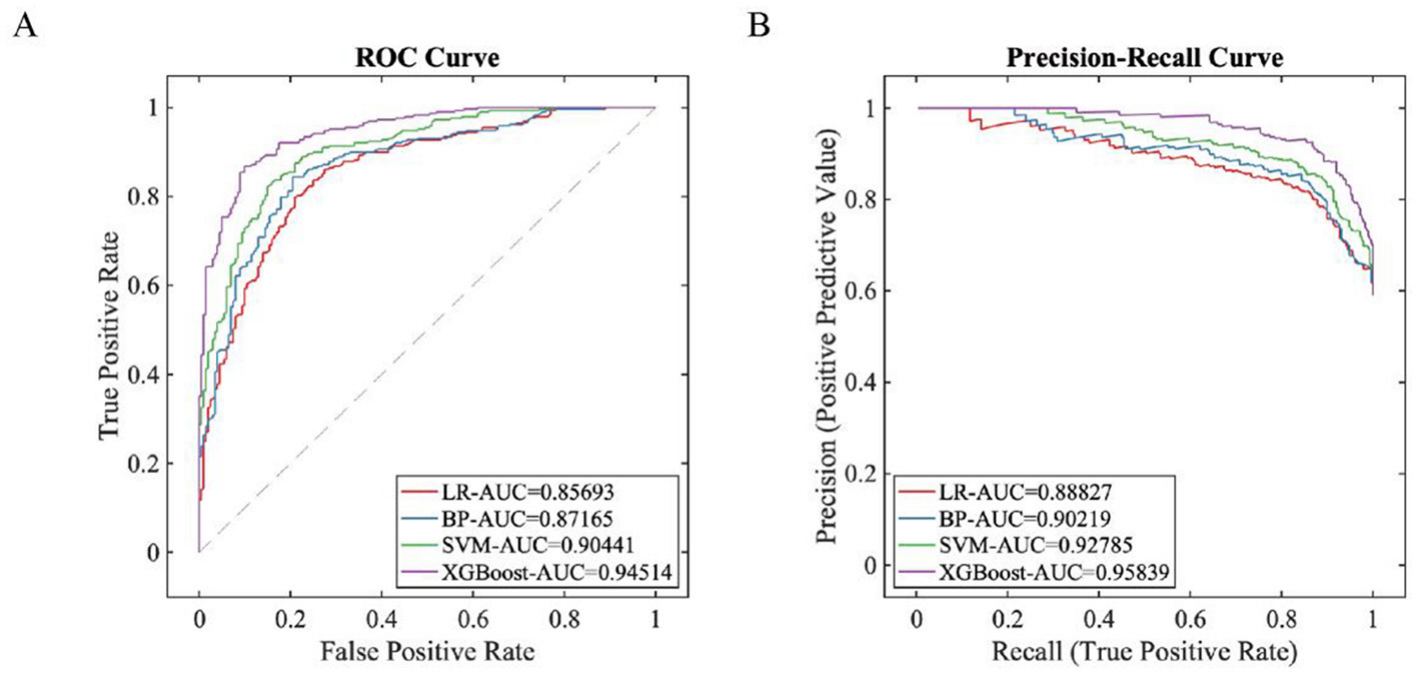
Training status of each model on the training set. **(A)** ROC Curve; **(B)** PR Curve.

Through stratified random sampling, 80% of the dataset was allocated as the training set (*n* = 433) while the remaining 20% formed the independent testing set (*n* = 108). Within the training set, we performed 5-folds cross-validation using IGKSO to identify optimal feature subsets and hyperparameter configurations. The results of the model training showed that the synchronous optimization prediction model with XGBoost as the base learner performed the best (Table 3, Figure 4). XGBoost was ultimately determined as the base learner, with the hyperparameter combination set as: learning_rate = 0.12, max_depth = 8, min_child_weight = 4, subsample = 0.85, colsample_bytree = 0.75, reg_alpha = 0.05, reg_lambda = 1.2 356. The finally selected features included: TUG time, WOMAC pain score, HADS anxiety score, knee extensor moment, age, sex, KL grade, and BMI.

**Table 3:** Comparison of cross-validation results for synchronously opptimized machine learning models.

**Base learners**	**PRE**	**SEN**	**SPE**	**ACC**	**F1**	**ROC-AUC**	**PR-AUC**
LR	0.7177	0.9271	0.4750	0.7418	0.8091	0.8569	0.8883
BP	0.8121	0.8854	0.7050	0.8115	0.8472	0.8717	0.9022
SVM	0.8168	0.9132	0.7050	0.8279	0.8623	0.9044	0.9279
XGBoost	0.8338	0.9410	0.7300	0.8545	0.8842	0.9451	0.9584

### 3.4 Model testing

**Figure 5:**
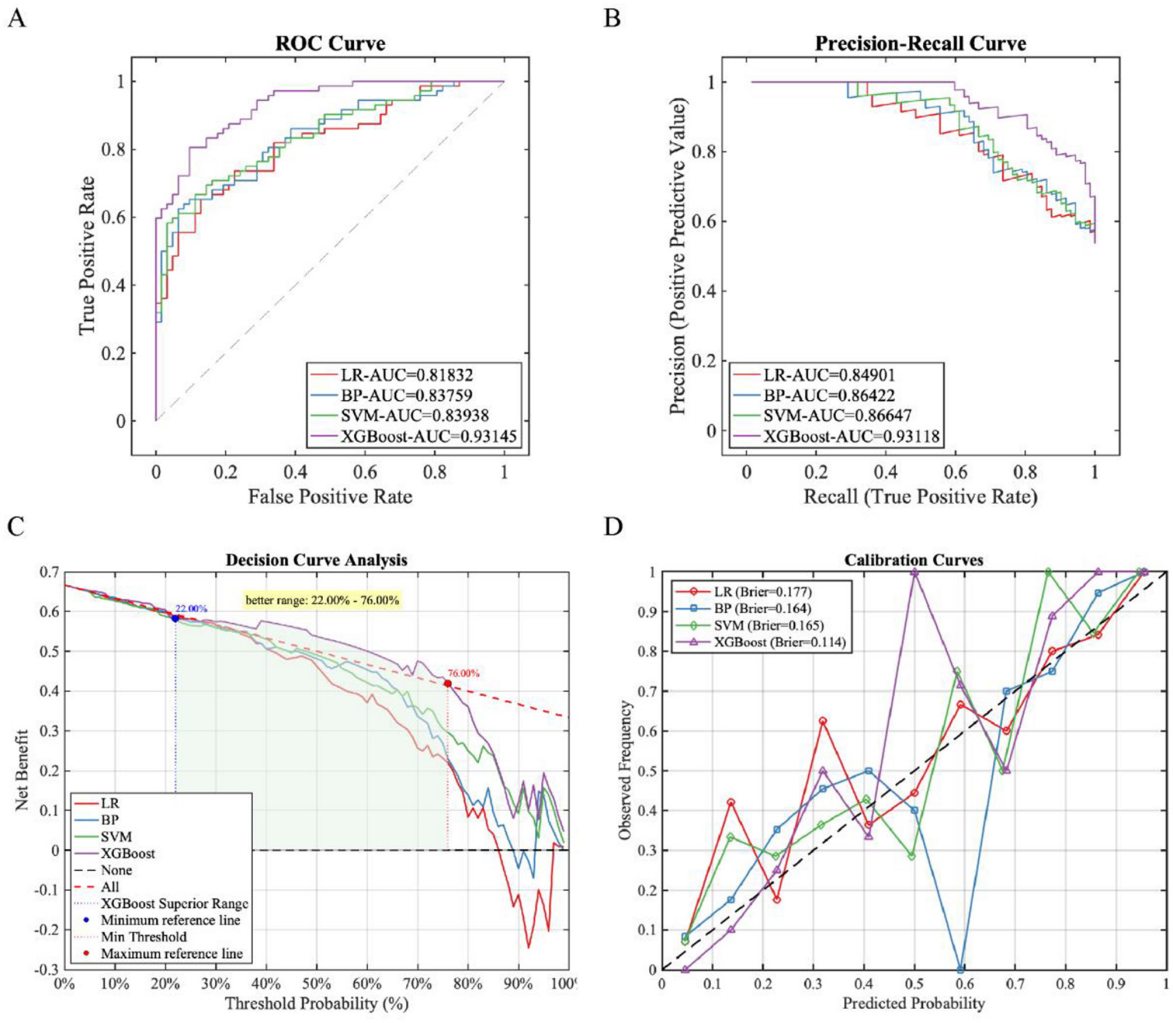
Predictive performance of each model on the test set. **(A)** ROC Curve; **(B)** PR Curve; **(C)** DCA curve; **(D)** Calibration curve.

The independent testing set (*n* = 108) was used to evaluate the generalization ability of each model. The results showed that the synchronous optimization prediction model with XGBoost as the base learner performed the best (Table 4, Figure 5). Decision curve analysis (Figure 3C) demonstrated that applying the XGBoost prediction model in the test set provided greater clinical net benefit compared to other methods across the 22%−76% risk threshold range. This model's net benefit curve maintained high levels with minimal fluctuations over this broad threshold probability interval, indicating excellent generalization capability and stable predictive performance. Calibration curve analysis (Figure 3D) confirmed the XGBoost model's predictive calibration significantly outperformed others, achieving the lowest Brier score (0.114) in the test set.

**Table 4:** Performance of various base learners based on synchronous optimization on the test set.

**Base learners**	**PRE**	**SEN**	**SPE**	**ACC**	**F1**	**ROC-AUC**	**PR-AUC**
LR	0.7342	0.8056	0.6613	0.7388	0.7682	0.8183	0.8490
BP	0.7634	0.9861	0.3889	0.7870	0.8606	0.8376	0.8642
SVM	0.6854	0.8472	0.5484	0.7090	0.7578	0.8394	0.8665
XGBoost	0.7692	0.9722	0.6613	0.8284	0.8589	0.9315	0.9312

### 3.5 Machine learning interpretability analysis

**Figure 6:**
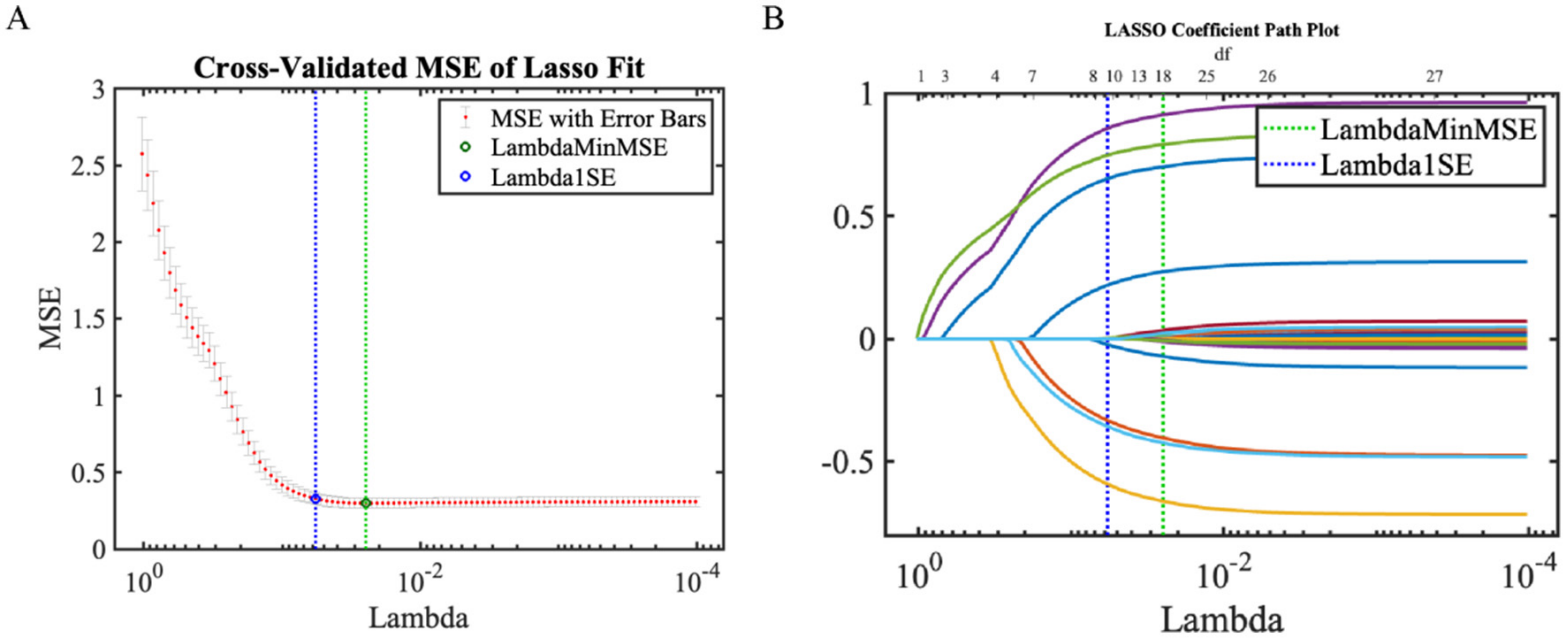
LASSO regression results. **(A)** LASSO trajectory plot; **(B)** LASSO cross-validation fitting plot.

LASSO regression was employed to conduct feature selection on the training set data (Figure 6), validating the effectiveness of feature screening by the synchronous optimization prediction model (XGBoost-based learner). LASSO selected variables within one standard error of the minimum MSE in the sparse model (Lambda1SE), screening out 8 variables that encompassed all features selected by the synchronous optimization prediction model. The eight features determined by the synchronous optimization prediction model: TUG time, HADS anxiety score, WOMAC pain score, sex, knee extensor moment, age, BMI, and KL grade, showed significant differences (*P* < 0.05) between the two patient groups. Details are presented in Table 5.

**Table 5:** Univariate analysis.

**Variables of interest**	**CAF group (*n* = 360)**	**No CAF group (*n* = 181)**	**Statistics value**	***P***
Age (Year), Mean ± SD	67.35 ± 14.23	61.85 ± 13.77	4.288	< 0.001
BMI (kg/m^2^), Mean ± SD	24.97 ± 3.78	23.12 ± 3.15	5.668	< 0.001
**Sex**, ***n*** **(%)**				
Male	78 (21.67)	86 (47.51)	38.091	< 0.001
Female	282 (78.33)	95 (52.49)		
Knee extensor moment (Nm/kg), Mean ± SD	1.05 ± 0.32	1.45 ± 0.41	12.449	< 0.001
TUG time (seconds), Mean ± SD	14.52 ± 3.85	10.11 ± 2.46	14.034	< 0.001
**KL classification**, ***n*** **(%)**				
Grade 2	202 (56.11)	130 (71.82)	12.542	< 0.001
Grade 3	158 (43.89)	51 (28.18)		
WOMAC pain score, Mean ± SD	6.45 ± 2.01	4.73 ± 1.24	10.545	< 0.001
HADS anxiety score, Mean ± SD	10.83 ± 3.31	9.11 ± 2.59	6.112	< 0.001

**Figure 7:**
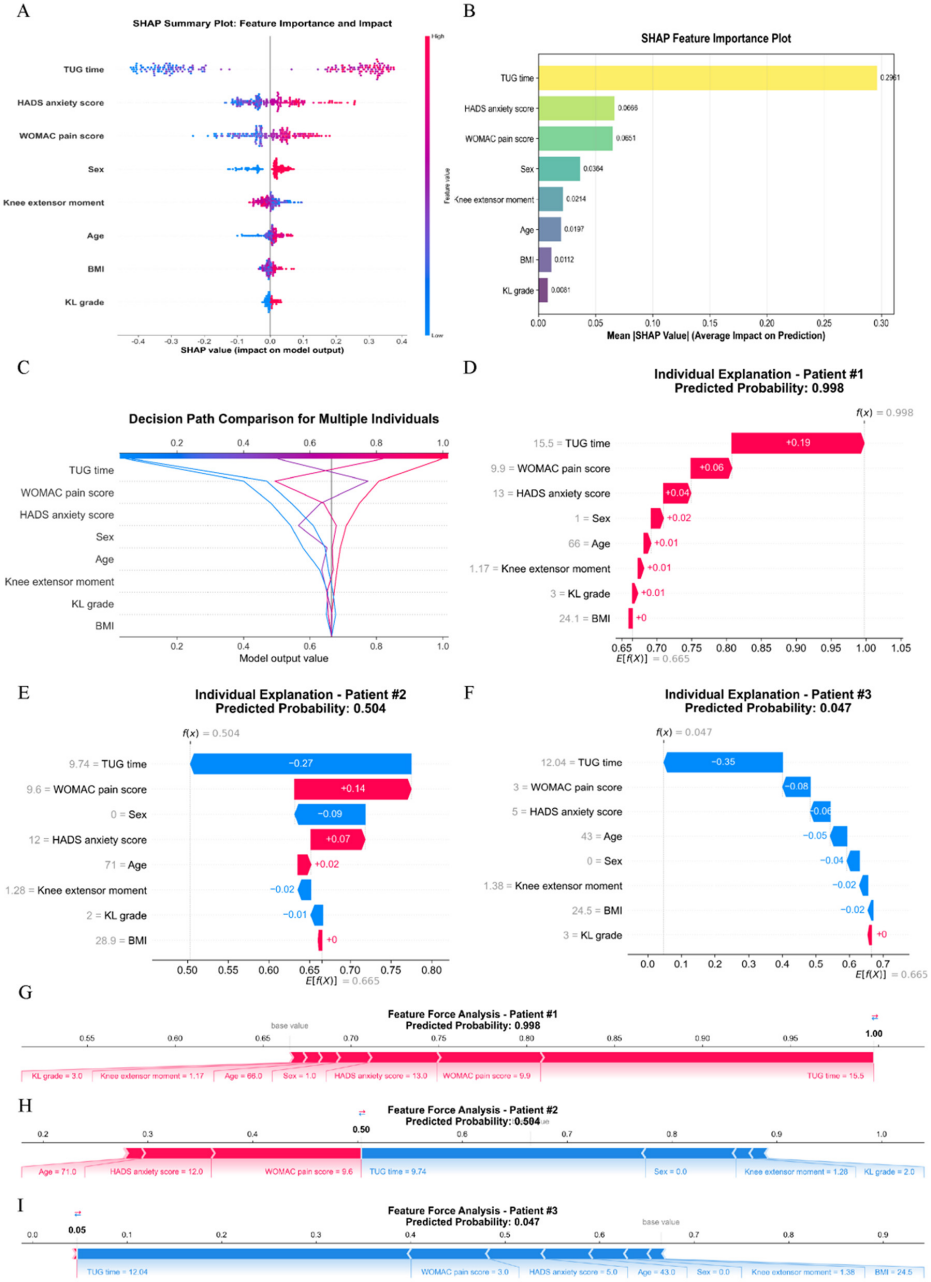
Overall SHAP value comparison of key indicators. **(A)** SHAP summary plot; **(B)** SHAP feature importance plot; **(C)** The decision path plot compares decision pathways across multiple patients, demonstrating how different feature combinations lead to varying prediction outcomes. The horizontal axis shows predicted probabilities, the vertical axis lists features, and the curved pathways trace decision routes from baseline values to final predictions; **(D-F)** Waterfall plots illustrate the cumulative contribution process of each feature to individual patient predictions. The baseline value represents the model's average prediction for all patients, while feature contributions show how each feature affects the final prediction (red indicating increased risk, blue indicating decreased risk). The sum of all feature contributions yields the final predicted value; **(G-I)** Force plots visually demonstrate how each feature “pushes” predictions toward higher or lower risk directions. Red arrows indicate features pushing predictions toward higher risk, blue arrows indicate features pushing toward lower risk, with arrow length representing the magnitude of influence.

Analysis of SHAP Results: Based on SHAP analysis (Figures 7A, B), features were ranked by importance as follows: TUG time, HADS anxiety score, WOMAC pain score, sex, knee extensor moment, age, BMI, and KL grading. By comparing decision paths across risk-stratified patients (Figure 7C), systematic differences in feature combinations emerged between high-risk and low-risk cohorts. The pronounced rightward shift in high-risk patients' paths indicated synergistic effects of multiple risk factors.

**Figure 8:**
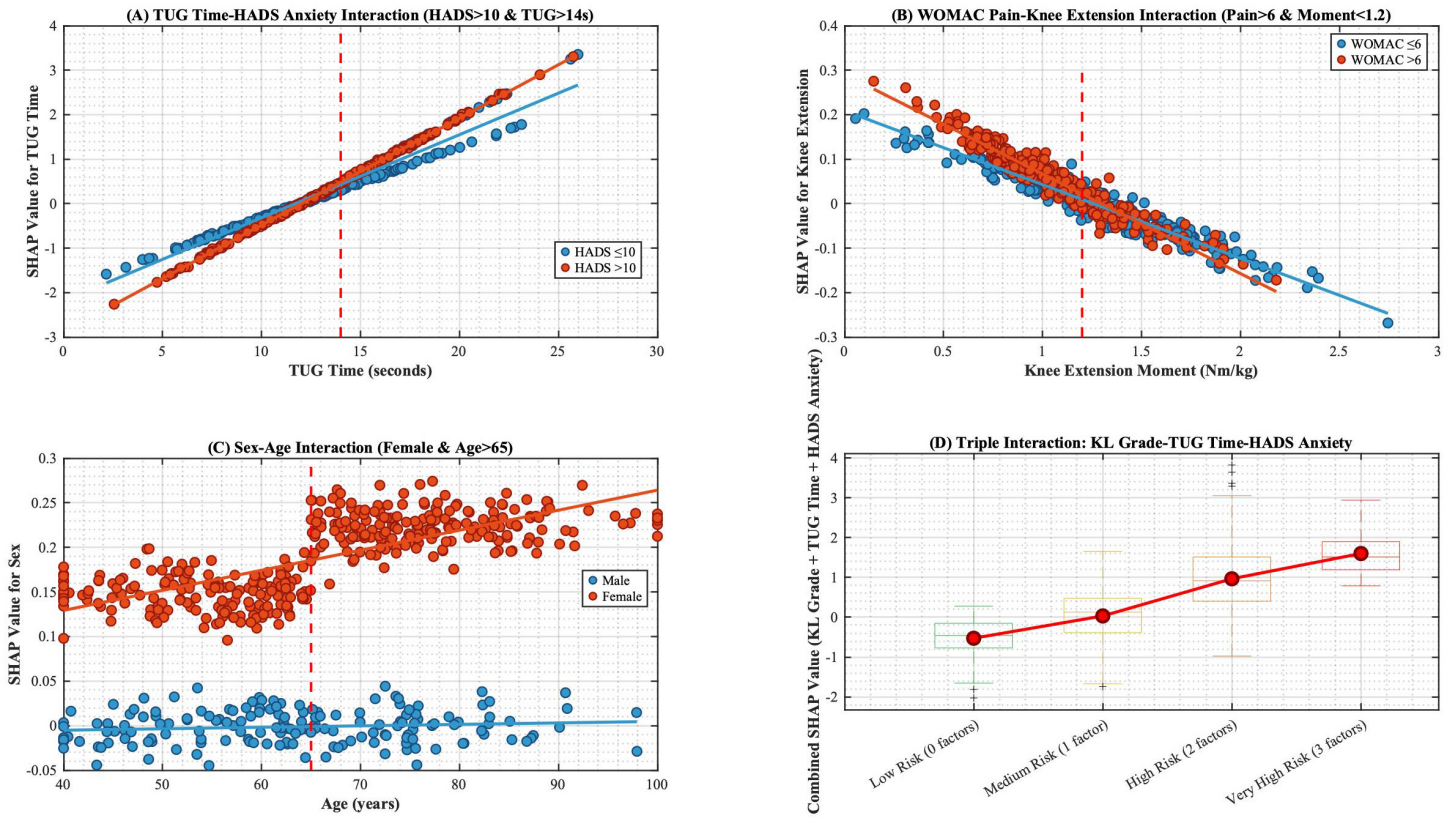
SHAP Interaction analysis between key indicators. SHAP Interaction Results (in this figure): **(A)** TUG time significantly increases the risk of fall concern, particularly in patients with HADS anxiety scores >10 (red lines indicate elevated SHAP values when TUG time exceeds 14 s in the high-anxiety group). **(B)** High WOMAC pain scores synergistically interact with low knee extensor torque to amplify risk (red lines demonstrate steeper negative slopes, indicating greater impact of low muscle strength when pain is severe). **(C)** Female patients exhibit higher fall concern risk, which escalates when age exceeds 65 years (red lines mark elevated SHAP values in older adult females), demonstrating significant sex-age interactions. **(D)** The triple-high-risk combination (KL grade 3 + TUG >14 s + HADS >10) yields the highest SHAP values, necessitating reinforced fall prevention interventions.

Case-Specific Interpretations:

(1) High-risk patient (Case 1, Figures 7D, G): A 66-year-old female with KL grade 3, TUG time of 15.5 s, WOMAC pain score of 9.9, HADS anxiety score of 13, and knee extensor torque of 1.17 Nm/kg exhibited a 99.8% predicted probability of CAF occurrence. SHAP analysis identified prolonged TUG time as the primary risk driver (highest positive SHAP value), compounded by elevated WOMAC pain and HADS anxiety scores. Weaker knee extensor torque further amplified risk. This highlights functional mobility impairment, severe pain, and high anxiety as critical risk factors in older adult females.
(2) Medium-risk patient (Case 2, Figures 7E, H): A 71-year-old male (KL grade 2) with TUG time 9.74 s, WOMAC pain 9.6, HADS anxiety 12, and knee torque 1.28 Nm/kg had a 50.4% CAF risk. While elevated pain/anxiety scores indicated risk, shorter TUG time (indicating better functional mobility) and protective factors (male sex, KL grade 2) mitigated the overall risk. This demonstrates how feature interactions—not isolated high-risk factors—dictate final predictions.
(3) Low-risk patient (Case 3, Figures 7F, I): A 43-year-old male (KL grade 3) with TUG time 12.04 s, WOMAC pain 3.0, HADS anxiety 5, and knee torque 1.38 Nm/kg had merely 4.7% CAF risk. Low WOMAC pain and HADS anxiety scores generated strong protective effects (highest negative SHAP values). Despite severe radiographic OA (KL grade 3), youth, minimal pain/anxiety, and robust muscle function collectively predicted low risk. This confirms that adequate psychological/physical function may override structural damage in CAF risk stratification. The SHAP interaction results for the variables are shown in Figure 8.

## 4 Discussion

This study employed machine learning models to construct a prediction model for CAF, ultimately selecting XGBoost as the base learner. The superior performance of the XGBoost model stems from its inherent suitability for clinical prediction tasks involving complex feature interactions. Its tree-based architecture excels at capturing nonlinear relationships and interactions among biomechanical, psychological, and demographic factors—key characteristics of CAF pathophysiology. Furthermore, its built-in regularization mechanism likely contributes to robust generalization on independent test sets, preventing overfitting commonly seen in more complex models (e.g., neural networks) with limited sample sizes. The eight variables ultimately identified—TUG time, HADS anxiety score, WOMAC pain score, sex, knee extensor torque, age, BMI, and KL grade—comprehensively map onto the multidimensional assessment framework of OARSI fall risk guidelines (1). Within the biomechanical domain, TUG time emerged as the primary predictor, with its threshold effect particularly pronounced due to gait adaptation dysfunction resulting from compromised joint stability (23). Concurrently, the synergistic worsening effect between low knee extensor torque and high WOMAC pain scores reflects pain's inhibitory impact on neuromuscular control (24). In the psychological domain, the interaction between HADS anxiety scores and TUG time reveals bidirectional amplification (25). Within the socio-demographic domain, the sex-age interaction proved especially significant, attributable to combined effects of estrogen decline-induced muscle mass loss and shifting social roles (26). KL grade, as a structural damage marker, amplifies negative effects across domains by accelerating proprioceptive dysfunction (23). While CAF and actual fall risk are distinct constructs, they exhibit bidirectional interactions in KOA populations. Biomechanical impairments directly heighten physiological fall susceptibility due to gait instability and decreased knee extensor torque. Concurrently, elevated CAF further restricts mobility through kinesiophobia, exacerbating muscle deconditioning and balance deficits. This creates a self-perpetuating cycle wherein physiological risk amplifies psychological distress (CAF), which in turn worsens physical capacity and increases actual fall incidence. Although CAF is an independent predictor of functional decline impacting of KOA patients, we recognize the clinical value of predicting objective falls. Our model identifies high-risk subgroups likely to benefit from preventive interventions (e.g., targeted strength training), thereby indirectly mitigating actual falls by breaking this cycle.

In terms of predictive weight, TUG time contributed most substantially to CAF risk. This phenomenon profoundly illustrates how declining functional mobility fuels psychological impairment through the “kinesiophobia-activity avoidance” cycle—as joint dysfunction reduces movement efficiency, catastrophic perceptions of fall consequences reinforce activity avoidance behaviors, accelerating muscle disuse and balance deterioration (27, 28). Notably, anxiety exerted catalytic effects: within the HADS>10 group, identical TUG time increases produced 1.8-folds greater CAF risk elevation vs. the low-anxiety group, confirming anxiety's role in amplifying environmental threat perception to exacerbate activity restriction (29, 30). Regarding gender disparities, older adult females demonstrated particular vulnerability, exhibiting significantly higher CAF risk than age-matched males (31). This difference arises from intersecting pathophysiological mechanisms: estrogen decline accelerates muscle protein breakdown via NF-κB pathway activation, causing faster quadriceps strength loss (32), while psychosocial studies indicate solitary older adult females face 2.3-folds greater fall-related concerns due to inadequate immediate care support (33). These intertwined biological and social factors establish this cohort as a CAF intervention priority.

Interaction analysis revealed marked multi-factor synergies in CAF risk development: prolonged TUG (>14s) and high anxiety (HADS > 10) interacted additively, with SHAP values sharply rising in high-anxiety patients (red lines), indicating anxiety amplifies threat perception and neuromuscular inhibition to worsen balance deficits from functional decline; high WOMAC pain and low knee torque (< 1.3 Nm/kg) formed synergistic negative slopes (red lines), confirming pain-induced central sensitization inhibits α-motoneuron recruitment causing neuromuscular decoupling (34); peak SHAP values in females (>65 years) revealed bio-social interactions, where estrogen-related muscle loss combines with social role transitions to amplify sex-age risks; the triple-risk combination (KL3 + TUG > 14s + HADS > 10) surpassed single-factor effects, mechanistically originating from combined joint structural damage compromising proprioception, functional mobility deficits, and anxiety-driven motor inhibition. Significantly, pain-torque imbalance caused more pronounced neuromuscular dysfunction in highly anxious individuals, while younger males maintained low risk despite KL3 damage through optimal psychological and muscular function—validating that biomechanical-psychological-social dynamics determine ultimate risk stratification.

This study has limitations. First, its retrospective design may introduce selection bias potentially affecting result accuracy. Second, while our optimized machine learning model achieved predictive success, further validation is required to ensure reliability. The retrospective approach and unimodal data limit granular assessment of activity-specific concerns—a strength of standardized tools like FES-I. Finally, clinical translation faces constraints as the MATLAB-based system impedes deployment in standard hospital environments lacking this proprietary platform. Critically, we are collaborating with med-tech partners to migrate to web-deployable frameworks (e.g., JavaScript/Python), enhancing accessibility. Future research should prioritize: (1) prospective designs minimizing bias; (2) expanded samples ensuring model generalizability; and (3) incorporation of additional clinical indicators optimizing predictive utility. Moreover, treatment-response heterogeneity was unexplored - future studies should examine whether interventions like laser acupuncture show differential effects across risk strata (35). We acknowledge that predicting actual falls offers direct clinical utility, and our team is actively developing models incorporating prospective fall-event tracking and inertial measurement unit (IMU)-derived gait variability metrics. Currently, we are validating algorithms using accelerometer data from KOA cohorts to quantify real-time balance loss during daily activities, with preliminary results expected in 2027. The optimized framework from this study—particularly its risk stratification capability—provides a foundation for translating CAF prediction into tangible fall-risk reduction. Subsequent studies will report predictive performances for actual falls alongside CAF outcomes, enhancing preventive precision.

## 5 Conclusion

This study developed an XGBoost model integrating biomechanical, psychological, and socio-demographic variables for precise CAF prediction in KOA. TUG time, HADS anxiety, and KL grade synergistically marked high risk, with the triple-risk combination elevating CAF probability to 99.8%. Conversely, structural damage patients preserved low risk through optimal physical/psychological reserves, enabling stratified interventions. Older adult females require focused management of estrogen-related muscle loss and social isolation risks. Future work will validate model generalizability in prospective cohorts and develop cloud-based tools for clinical translation.

## Data Availability

The raw data supporting the conclusions of this article will be made available by the authors, without undue reservation.
